# Dataset on the use of biostimulants in strawberry cultivation under tropical conditions

**DOI:** 10.1016/j.dib.2025.111445

**Published:** 2025-03-07

**Authors:** Maria Celina Villagra Luraschi, Noelia Isabel Godoy Medina, Victoria Rossmary Santacruz Oviedo, Romina Burgos Rotela, Cipriano Ramón Enciso-Garay

**Affiliations:** a Universidad de São Paulo, Escola Superior de Agricultura (USP/ESALQ) Piracicaba - SP, 13418-900, Brasil; b Universidad Nacional de Asunción, Facultad de Ciencias Agrarias (UNA/FCA), Campus UNA 2169, San Lorenzo Py-11001-900, Paraguay

**Keywords:** Fragaria × ananassa, Perfomance, Total sugars, Fruits quality, Yield

## Abstract

In tropical conditions, due to high temperatures, the use of plant management practices is necessary to increase productivity. For strawberry production, biostimulants can promote an increase fruit quality and production. These substances can promote plant growth, increase stress tolerance, promote root growth and nutrient absorption, increase stress tolerance and improve fruit quality. In some Latin American countries, including Paraguay, the strawberry production system is carried out by family farming, with poor management of seedlings, production technology, health problems and lack of access to new varieties. The objective of this work was to determine how different doses of biostimulants affect seven varieties of strawberry. The experiment was conducted at the Universidad Nacional de Asunción, Facultad de Ciencias Agrarias (UNA/FCA), during March–July 2022. The treatments consisted of a combination of seven varieties of strawberry with three levels of biostimulants. The varieties were Sabrina, Camino Real, Festival, Sweet Charlie, Dover, Francesita, Early Bright and the doses of biostimulant applied were 0 ml/L, 1 ml/L and 2 ml/L. The experimental design was subdivided into plots with a bifactorial arrangement (7x3), where factor I was the three doses and factor II was the seven varieties. Each treatment had four repetitions, and each experimental unit consisted of four plants. Evaluated parameters included, total and commercial fruit number per plant, total and commercial fruit mass, total and commercial yield per square meter, diameter and length of the fruit, content of soluble solids totals, titratable acidity, pH and fruit firmness. Data were analyzed using analysis of variance (ANOVA) followed by Tukey´s test at the 5 % significance level. Dover and Festival varieties achieved excellent productivity (4.63 and 3.17 kg m² respectively), regardless of the treatment doses.

Specifications Table

**Table:** 

Subject	*Agricultural Science*
Specific subject area	*Horticulture*
Type of data	Table, Figure
. Data collection	In collaboration with a team of researchers, the evaluation work of seven varieties of strawberry grown in the experimental field of the Universidad Nacional de Asunción, Facultad de Ciencias Agrarias (UNA/FCA) the data was collected in the year 2022. Strawberry varieties Dover, Sweet Charlie, Festival, Sabrina, Camino Real, Early Bright and Francesita were used. In total there were 21 treatments where factor 1 was the strawberry varieties and factor 2 was the biostimulant doses (0 ml/L, 1.0 mL/L and 2.0 mL/L). The commercial biostimulant used was topseed gram, applied by foliar spraying twice a month until the end of the plant cycle. The experimental design used was in subdivided plots with a bifactorial arrangement 7 × 3 with four repetitions. Irrigation was applied by drip, as well as nutrients via fertigation. To evaluate the effect of the treatments, four plants were taken per experimental unit and the harvests were carried out every two days, the variables studied being: total and commercial number of fruits, total and commercial mass of fruits, total and commercial productivity of fruits. The postharvest were collected at the UNA/FCA Postharvest and Quality Laboratory. Ten fully red fruits from each treatment were randomly selected, and fruit length, diameter of fruit, shape, firmness, titratable acidity, pH, and total soluble solids were measured The data were arranged in excel form and subjected to analysis of variance (ANOVA) and in case of finding significant statistical differences, the data were subjected to the comparison of means by the Tukey test at 5 % probability of error. The AGROSAT program was used.
Data source location	Universidad Nacional de Asunción, Facultad de Ciencias Agrarias, Campus Universitario, San Lorenzo, Central, Paraguay, coordinates 25° 20′17 '' S and 57° 31′06 '' W and at 120 m above sea level :www.agr.una.py
Data accessibility	Repository name: Dataset on the use of biostimulants in strawberry cultivation under tropical Raw and statistics data in Mendeley Repository Data identification number: doi: 10.17632/jb49jwmdwp.3 Direct URL to data: https://data.mendeley.com/datasets/jb49jwmdwp/3.
Related research article	none.

## 1 Value of the Data

**Fig. 1:**
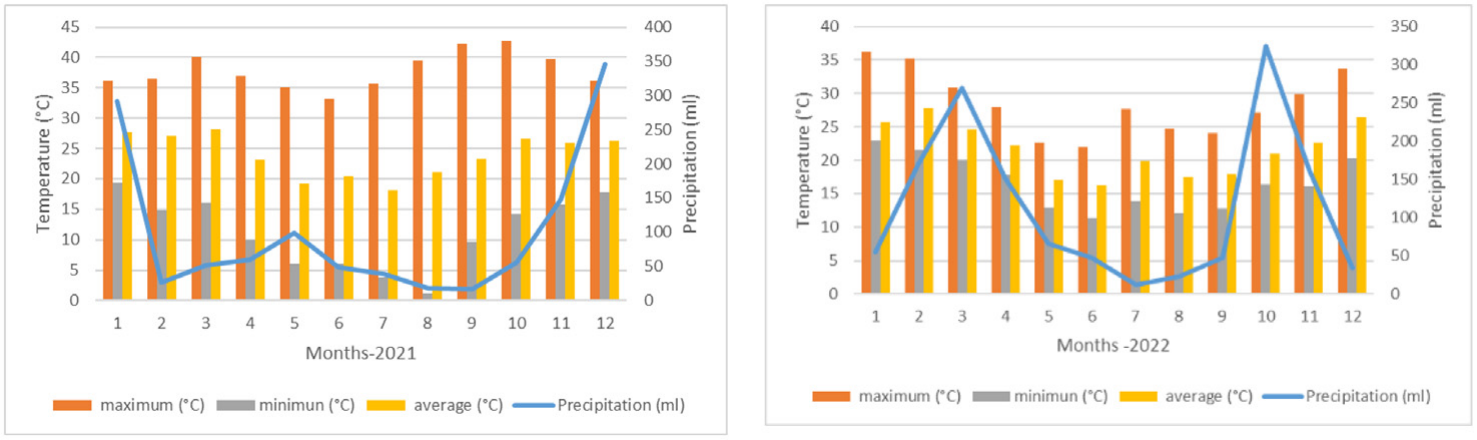
Temperature (°C) and Precipitation (mm) years 2021-2022 (DINAC 2022) [17].
• The set of data presented on the use of biostimulants, comparisons of varieties and climates can be useful for researchers and technicians when they are looking for alternatives that contribute to improving production in the field. This set of data can generate adjustments and/or alternatives in the application of this type of bio input, in addition to the fact that the strawberry is very sensitive to chemical fertilization, in addition to unfavorable climatic conditions. Therefore, there is important information on varieties, climate behavior during field production and the effects of biostimulant doses, which can contribute to the improvement of production with reduced costs and increase the benefits that this crop offers to the population*.*

• In most Latin American and Caribbean countries, strawberry production is carried out through traditional family farming with limited technological management and poor bed preparation (field production). There is no cultivar renewal, as seen in Paraguay, where farmers continue using only one or two varieties, such as Dover and Sweet Charlie, for periods exceeding 20 years. This lack of renewal leads to phytosanitary problems for horticulturists and increases production costs.
• On the other hand, the maximum temperatures (Fig. 1) are high in the production periods (late summer‒autumn‒winter) of this species, and the transplant season can be delayed between two and three weeks. This situation leads to late transplantation and limits the development of strawberry plants, both root and aerial, which reach the time of floral differentiation without a sufficient number of leaves and consequently with the production of small fruits and low yields. The national production is 361 g plant^-1^ (CAN 2022) [1], with the productive potential of the species being between 800 and 1200 g per plant (JICA 2002) [2]. These factors drive the ongoing efforts to enhance crop productivity and quality

## 2 Background

The introduction of strawberry cultivation demands intensive use of the soil, and the impact of the use of fertilizers and pesticides negatively affects the environment and health of consumers. The use of biostimulants can minimize the negative impact, improve fruit quality, and plant productivity (Soltaniband 2022) [3], Kilic (2024) [4].

A plant biostimulant is any substance or microorganism that is applied to plants with the aim of improving nutritional efficiency, tolerance to abiotic stress and/or quality characteristics of the crop, regardless of its nutrient content (du Jardin 2015[5], Tarafdar 2022) [6], and its physiological effects depend on its composition (Parađiković 2018) [7].

In strawberry, biostimulants are used before transplantation, in seedlings, (Kirschbaum et al. 2019) [8] (Hassan et al. 2021) [9], before flowering, during flowering, at the onset of fruiting and fruiting (Bogunovic et al. 2015 [10], Spoljarević et al. 2010) [11].

The positive effects of the application of biostimulants include improvements in growth and productivity (Ashour 2023) [12], greatest leaf areas (Kouam et al. 2024) [13], increases in nutritional quality (Chandramohan 2020 [14] and, resistance to low temperatures (Bogunovic et al. 2015) [9].

In a study using soilless cultivation, Ranasingha et al. (2024) [15], observed a significant increase in the number of marketable fruits (55 %) and average fruit weight (56 %) compared to the control group of strawberries. Drobek et al. (2024) [16], reported improved taste and increased nutritional value in strawberries, including a 14 % average increase in soluble solid content (SSC). Kilic (2024) [4], found that combining organic fertilizers and humus in organic strawberry production increased yield, fruit quality, plant growth, and nutrient content.

The objective of this work was to evaluate the productivity and quality of strawberry cultivars with different levels of biostimulant and its effect on the quality of strawberry

## 3 Data Description

### 3.1 Temperature and precipitation

Fig. 1 presents data on the monthly averages of temperature and precipitation for the years 2022 and 2021.

### 3.2 Total number of fruits

The statistical analysis revealed significant differences in the cultivar factor, the dose factor and the cultivar interaction according to the dose of biostimulant (Table 1).

**Table 1:** Mean squares of the analysis of variance of the agronomic variables of strawberry varieties as a function of biostimulant levels.

Variation source	Mean squares						
	Agronomic variables						
	Df	TNF (fruits plant ^-1^)	CNF (fruits plant ^-1^)	TMF (g plant ^-^^1^)	CMF (g plant ^-1^)	TYield (kg/m^2^)	CYield (kg m^2^)
Block	3	3.370 ^ns^	2.022 ^ns^	267.4 ^ns^	367.09 ^ns^	0.0667 ^ns^	0.094 ^ns^
Cultivar	6	497.06^⁎⁎^	596.46^⁎⁎^	65234.8^⁎⁎^	77511.0 ^⁎⁎^	16.703^⁎⁎^	19.81^⁎⁎^
Error a	18	1.70	2.64	1163.13	1638.2	0.2970	0.419
Biostimulant	2	91.175^⁎⁎^	108.88^⁎⁎^	15577.7^⁎⁎^	18200.7^⁎⁎^	7.957^⁎⁎^	4.65^⁎⁎^
Cultivar x biostimulant	12	51.20 ^⁎⁎^	61.75^⁎⁎^	8380.4^⁎⁎^	8645.70^⁎⁎^	25.746^⁎⁎^	2.21^⁎⁎^
Error b	42	2.680	2.75	617.5	556.16	0.158	0.142
CV (%) (cultivar)	-	8.48	12.10	20.6	26.76	20.61	26.75
CV(%)biostimulant		10.64	12.35	15.0	15.59	15.03	15.58
Mean	-	15.38	14.44	165.3	151.22	2.64	2.41

CV Coefficient of Variation, df (degrees of freedom) TNF (Total number of fruits) CNF (Commercial number of fruits) TMF (Total mas of fruits) CMF (Commercial mass of fruits) TYield (Total yield) CYield (Commercial yield)). * ,** : significance at P≤0.05, P≤0.01and P≤0.001, respectively*.*

With respect to the cultivar factor, in most cases, the control without biostimulant application presented higher values. Camino Real and Festival were the only ones to present significant differences with the use of both doses of biostimulant used (Table 2).

**Table 2:** Total and commercial number of fruits of strawberry cultivars based on biostimulant levels.

Cultivars								
Biostimulant	Sabrina	Camino Real	Festival	Sweet Charlie	Dover	Francesita	Early Bright	dose
Total number of fruits (plant fruits^-1^)								
0 ml L^-1^	8.08 Ae	10.13 Be	13.83 Cd	20.33 Ab	33.08 Aa	19.75 Abc	16.49 Acd	17.38 A
1 ml L^-1^	8.75 Ac	12.00 Bc	17.58 Bb	9.92 Bc	25.83 Ba	11.33 Bc	11. 74 Bc	13.88 B
2 ml L^-1^	8.58 Ade	13.38 Ac	21.63 Ab	11.83 Bcd	26. 50 Ba	13.81 Bc	8.50 Ce	14.89 B
$\bar{x}$ cultivar	8.47 e	11.84 d	17.68 b	14.03 c	28.47 a	14.97 e	12.24 d	
Commercial number of fruits (plant fruits^-1^)								
0 ml L^-1^	5.42 Ae	6.67 B e	12.67 Cd	19.17 Ab	31.75 Aa	18.50 A bc	15.24 A cd	15.63 A
1 ml L^-1^	5.08 Ae	10.88 Ac	16.67 Bb	6.58 Bde	24.75 B a	9.17 C cd	9.49 B cd	11.80 C
2 ml L^-1^	5.06 Ae	11.75 Ade	19.63 Ab	8.83 Bcd	25. 83 Ba	12.31 B c	6.83 B de	12.89 B
$\bar{x}$ cultivars	5.19 e	9.77 d	16.32 b	11.53 cd	27.44 a	13.33 c	10.52 d	

Same letters, uppercase in the columns and lowercase in the lines, do not differ by Tukey's test (*P* ≤ 0.05).

The interaction results revealed that the cultivar Dover (33.08 plant fruits^-1^), Sweet Charlie (20.33 plant fruits^-1^), Francesita (19.75 plant fruits^-1^) and Early Bright (16.49 plant fruits^-1^) responded with a greater number of fruits to the control with respect to the other doses. The second best response was obtained with the 2 ml/ L dose, followed by the 1 ml/L dose. Except for Early Bright, the second best dose was 1 ml/L. On the other hand, the Camino Real and Festival groups responded better to the 2 ml/L treatment, with 13.38 and 21.63 plant^-1^ fruits, respectively, than did the control group, which was lower (Table 2).

### 3.3 Total fresh mass of fruits per plant

The results revealed that there were significant differences in the total fresh mass of fruits for both factors and for the interaction (Table 1).

The average of the highest value of total fresh mass of fruits corresponds to the cultivar Dover, with 291.28 g plant^-1^. In second place, there are Festival with 209.31 g plant^-1^ and Francesita with 196.73 g plant^-1^. Early Bright had an average value of 158.08 g plant^-1^, which, in turn, is similar to that of Sweet Charlie, with 125.67 g plant^-1^. The lowest values for the total fresh mass of fruits were Camino Real and Sabrina, with 99.71 and 76.93 g plant^-1^, respectively (Table 3).

**Table 3:** Total and commercial mass of fruits per plant of strawberry varieties based on biostimulant levels.

Cultivars								
Biostimulant dose	Sabrina	Camino Real	Festival	Sweet Charlie	Dover	Francesita	Early Bright	$\bar{x}$ dose
Total fresh mass of fruits (grams plant^-1^)								
0 ml/L	71.58 Ae	78.38 Ae	156.42 Bd	186.3 Acd	351. 0 A a	272.0 Ab	226.7 Ad	191.76 A
1 ml/L	78.33 Bc	90.63 Abc	226.00 Aa	87.08 Bc	251.8 Ba	155.8 Bb	135.6 Bbc	146.46 B
2 ml /L	103.5 Bbc	130.1 Abc	245.50 A a	103.6 B bc	271.1 B a	162. 4 Bb	112.0 B bc	157.72 B
$\bar{x}$ cultivar	76.43 F	99.71 EF	209.31 B	125.67 DE	291.28 A	196.73 BC	158.08 CD	-
Commercial fruit mass (grams plant^-1^)								
0 ml/L	51.83 Ae	61.17 B e	148.7 Bd	168.2 A cd	349.2 A a	263.9 Ab	216.0 Abc	179.88 A
1 ml/L	44.83 Ad	85.8 ABbcd	220.1 Aa	61.83 B cd	249.3 B a	138.7 Bb	116.7 Bbc	131.04 B
2 ml/L	52.63 Ad	121.0 A bc	224. 8 Aa	224.9 A a	268.5 B a	154.2 Bb	96.0 Bbcd	142.76 B
$\bar{x}$ cultivars	49.76 e	89.35 de	197.88 b	104.08 de	289.0 a	185.61 bc	142.90 cd	-

The same letters, uppercase in the columns and lowercase in the lines, do not differ by the Tukey test (P ≤ 0.05).

Regarding the dose of biostimulant and its influence on the total fresh mass of fruits, the control presented a greater average. The cultivars presented the best response to the control treatment, followed by the 2 ml/L treatment and finally the 1 ml/L treatment. On the other hand, the Festival results were significantly different from those of the control (156.42 g plant^-1^), with greater total masses of 226 and 245 g of plant-^1^ per 1 ml and 2 ml, respectively (Table 3).

### 3.4 Commercial fresh mass of fruits per plant

In the statistical analysis, there were significant differences between cultivars, biostimulant dose, and in the cultivar × biostimulant dose interaction (Table 1).

The Dover cultivar had the best performance, reaching a yield of 289 g plant ^-1^, followed by the Festival cultivar, with a yield of 197.88 g plant^- 1^, which did not differ statistically from that of Francesita, with a yield of 185.61 g plant^-1^. Early Bright had an average value of 142.90 g plant^-1^, which statistically coincides with Sweet Charlie, with 104.08 g plant^-1^, and Sabrina presented the lowest value, with 49.76 g plant ^-^^1^ (Table 3).

In terms of dose, the control had the greatest average of 179.88 g plant^-1^, followed by 2 ml/L and 1 ml/L, which did not differ from each other, with averages of 142.76 g plant^-1^ and 131.04 g plant^-1^, respectively (Table 3).

In terms of the interaction between cultivar and dose, all cultivars presented a relatively high commercial mass of fruits without the application of biostimulant, and the second-best response to dose was recorded at a dose of 2 ml/L, with the exception of Early Bright. In addition, for the Camino Real and Festival varieties, with a dose of 2 ml/L, they presented a better response (Table 3).

### 3.5 Total and commercial yields (kg m^-2^)

Total yield showed significant differences among cultivars, biostimulant doses, and their interactions (Table 1).

Dover was the highest yielding cultivar, producing an average of 4.66 kg/m^2^, followed by Festival with 3.35 kg/m^2^ and Francesita with 3.15 kg m^-2^. Intermediate yields were observed in Early Bright (2.53 kg/ m^2^), Sweet Charlie (2.01 kg/m^2^), and Camino Real (1.60 kg/m^2^). Sabrina showed the lowest performance with only 1.22 kg/m^2^ (Table 3).

For the biostimulant dose factor, the highest mean corresponded to the control (3.07 kg/m^2^), followed by the doses of 2 ml/L and 1 ml/L dose with averages of 2.52 kg/m^2^ and 2.34 kg/m^2^, respectively (Table 3).

In terms of the interaction, the cultivars Dover, Sweet Charlie, and Francesita and Early Bright presented better yields than did the control (0 ml/L) and, second, the highest dose used (2 ml/L). Camino Real and Festival achieved better performance with both doses of biostimulant and, finally, the control. On the other hand, Sabrina did not differ in terms of the doses applied (Table 3).

When the response of the cultivars to the different doses was compared, for the control, Dover had the best performance, with a value of 5.61 kg m^-2^, and Sabrina had the lowest yield, with a value of only 1.15 kg m^-2^. For the second best dose Dover responded well (4.34 kg m^-2^) during the Festival (3.93 kg m^-2^), but Sabrina responded well (1.25 kg m^-2^) (Table 4).

**Table 4:** Total and commercial yield of strawberry varieties based on biostimulant levels.

Biostimulant dose	Cultivars							
	Dover	Sweet Charlie	Sabrina	Francesita	Camino Real	Early Bright	Festival	$\bar{x}$dosis
Total yield (kg/m^2^)								
D1 (0 ml/L)	5.61 Aa	2.98 Acd	1.15 Ae	4.35 Ab	1.25 Be	3.63 Abc	2.50 Bd	3.07 A
D2 (1 ml/L)	4.03 Ba	1.39 Bc	1.25 Ac	2.49 Bb	1.45 ABc	2.17 Bbc	3.62 Aa	2.34 B
D3 (2 ml/ L)	4.34 Ba	1.66 Bbc	1.27 Ac	2.60 Bb	2.08 Abc	1.79 Bbc	3.93 Aa	2.52 B
$\bar{x}$ cultivar	4.66 a	2.01 de	1.22 f	3.15 bc	1.60 ed	2.53 cd	3.35 b	
Commercial yield (kg/m^2^)								
D1 (0 ml/L)	5.59 Aa	2.70 Acd	0.83 Ae	4.22 Ab	0.98 Be	3.46 Abc	2.38 Bd	2.88 A
D2 (1 ml/ L	3.99 Ba	0.99 Bcd	0.72 Ad	2.22 Bb	1.37 ABbcd	1.87 Bbc	3.52 Aa	2.10 B
D3 (2 ml/ L	4.30 Ba	1.31 Bcd	0.84 Ad	2.47 Bb	1.94 Abc	1.53 Bbcd	3.6 Aa	2.28 B
$\bar{x}$ cultivar	4.63 a	1.67 de	0.80 e	2.97 bc	1.43 de	2.29 cd	3.17 b	

The same letters, uppercase in the columns and lowercase in the lines, do not differ by the Tukey test (*P* ≤ 0.05).

For the biostimulant dose, the control showed the highest yield (2.88 kg/m²), which was different from the doses of 1 mL/L and 2 mL/L, which presented lower yields (2.10 and 2.28 kg/m² respectively (Table 5).

**Table 5:** Square means of variance analysis of fruit quality variables of strawberry cultivars based on biostimulant levels.

Source of variation	Square mean Fruit quality variables								
	df	FL (cm)	FD (cm)	TSS (°B)	df	FS	Firmnes	TA %	pH
Block	3	0.389^ns^	0.2434^ns^	0.37^n^	2	0.002^ns^	0.001^ns^	2.29 x 10^-7^^ns^	6.410^-4ns^
Cultivar	6	1.446**	0.6941*	3.97^⁎⁎^	6	0.018**	0.169**	0.006**	0.1001**
Error a	18	0.336	0.215	0.41	12	0.002	0.0004	9.9 x 10^-7 ns^	3.8.10^-4^
Biostimulant	2	2.149**	0.890*	2.76^⁎⁎^	2	1.36^ns^	0.132**	0.0003**	1.44^ns^
Cultivar*biostimulant	12	1.56**	0.75^⁎⁎^	0.51*	12	0.011*	0.138**	0.089**	0.019**
Error b	42	0.313	0.186	0.21	28	0.005	0.0004	9.9.10^-7^	9.210^-4^
CV (%) (cultivars)	-	18.40	17.56	10.4	-	4.37	2.68	0.25	0.56
CV(%) (Biostimulant)		17.76	16.36	7.48	-	5.92	2.81	0.25	0.86
Mean	-	3.15	2.64	6.19	-	1.19	0.75	0.39	3.5

CV (Coefficient of variation) df (degrees of freedom) FL(Fruit length) FD (fruit diameter) TSS (Total soluble solids) Fruit shape (FS) TA (Titratable acidity).

For the interaction between varieties and doses, the Dover variety showed a better response without biostimulant (0 mL), Festival responded with better performance to doses of biostimulants (1 and 2 mL), as well as the Camino Real variety showed a better response to higher doses (2 mL) (Table 5).

The analysis of variance (ANOVA) for the fruit quality parameters indicates, in relation to the varieties, significant effects (**) for almost all variables, such as fruit length (LF), total soluble solids (TSS), fruit shape (FF), firmness, titratable acidity (TA), pH, and significant (*) for fruit diameter (FD) (Table 5).

On the other hand, the biostimulant showed significant effects (**) for FL, SST, Firmness and AC, also being significant (*) for DF and without significant effects for FS and pH. The Cultivar*Biostimulant Interaction is significant (**) for FL, FD, Firmness, AC and pH and significant (*) for SST and FS (Table 5).

### 3.6 Diameter and length of the fruit

Significant differences in fruit diameter among cultivars were observed and fertilizer doses, as well as in their interaction (Table 5)

Among the cultivars, the highest average corresponds to Camino Real at 3.07 cm, similar to Early Bright (2.79 cm), Francesita (2.73 cm), Festival (2.65 cm) and Sweet Charlie (2.47 cm), and different from Dover (2.42 cm) and Sabrina (2.40 cm) (Table 6).

**Table 6:** Diameter and length of fruits of strawberry varieties based on biostimulant levels.

Biostimulant dose	Cultivars							
	Sabrina	Camino Real	Festival	Sweet Charlie	Dover	Francesita	Early Bright	$\bar{x}$ dose
Fruit diameter (cm)								
0 ml L^-1^	2.33 A b	4.38 A a	2.61 A b	2.47 A b	2.51 A b	2.78 A b	2.88 A b	2.85 A
1 ml L^-1^	2.34 A a	2.32 B a	2.76 A a	2.47 A a	2.31 A a	2.80 A a	2.76 A a	2.52 B
2 ml L^-1^	2.52 A a	2.51 B a	2.58 A a	2.49 A a	2.44 A a	2.61 A a	2.58 A a	2.57 B
$\bar{x}$ cultivar	2.40 b	2.47 ab	2.40 b	2.73 ab	3.07 a	2.79 ab	2.65 ab	
Fruit length (cm)								
0 ml L^-1^	2.91 A b	5.67 A a	3.24 A b	2.80 A b	3.04 A b	3.49 A b	3.14 A b	3.47 A
1 ml L^-1^	2.80 Aa	2.63 B a	3.37 A a	2.50 A a	2.90 A a	3.47 A a	3.02 A a	2.96 B
2 ml L^-1^	3.15 A a	2.97 B a	3.13 A a	2.74 A a	3.01 A a	3.17 A a	3.03 A a	3.03 B
$\bar{x}$ cultivar	2.95 b	3.76 a	3.24 ab	2.68 b	2.99 ab	3.38 ab	3.06 ab	

The same letters, uppercase in the columns and lowercase in the lines, do not differ by the Tukey test (P ≤ 0.05).

The greatest effect was the control (0 mL/L), with an average of 2.85 cm, followed by the biostimulant with 2 ml/L and 1 ml/L, with 2.57 and 2.52 cm, respectively (Table 6).

Fruit length varied significantly among cultivars, biostimulant doses, and their interaction (Table 6).

Among cultivars, Camino Real exhibited the highest mean fruit length (3.76 cm), similar to Francesita, Festival, Early Bright, and Dover (2.99–3.38 cm). These were statistically similar to the lower means observed for Sabrina (2.95 cm) and Sweet Charlie (2.68 cm) (Table 6).

For the dose factor, the mean fruit length was greater for the control (3.47 cm), being statistically different from both doses of biostimulant used (Table 6).

### 3.7 Total soluble solids and titratable acidity

With respect to the total soluble solids, there were significant differences in the cultivar factor, the dose factor and, the cultivar interaction according to the dose of biostimulant (Table 5).

For the cultivars, Dover, Sweet Charlie, Francesita, Early Bright and Festival presented the highest average values (between 6.06 °B and 6.83 °Brix), followed by Sabrina and Camino Real, with averages less than 5.42 and 5. 48 °Brix (Table 7).

**Table 7:** Content of soluble solids and titratable acidity of fruits of strawberry varieties based on biostimulant levels.

Biostimulant dose	Cultivars							
	Sabrina	Camino Real	Festival	Sweet Charlie	Dover	Francesita	Early Bright	$\bar{x}$ dose
Soluble solids content (° B)								
0 ml/L	5.21 A c	5.56 ABbc	5.98 AB abc	6.37 ABab	6.44 A ab	7.14 A a	6.75 AB a	6.20 B
1 ml /L	5.72 Aab	4.93 B b	5.42 B ab	6.03 B ab	6.35 A a	6.53 A a	6.18 B a	5.88 C
2 ml /L	5.32 A c	5.94 A bc	6.76 A ab	6.89 A ab	6.37 A abc	6.83 A ab	7.45 A a	6.51 A
$\bar{x}$ cultivar	5.42 b	5.48 b	6.06 ab	6.43 a	6.39 a	6.83 a	6.79 a	-
Tiritable acid (% citric acid)								
0 ml/L	0.33 C e	0.43 A c	0.38 C d	0.38 B d	0.32 C f	0.44 B b	0.44 A a	0.39 A
1 ml/L	0.42 A b	0.35 C f	0.45 B a	0.41 A c	0.38 A d	0.35 C e	0.35 B f	0.39 A
2 ml/L	0.40 B d	0.42 B c	0.47 A a	0.34 C f	0.35 B e	0.44 A b	0.34 C f	0.39 A
$\bar{x}$ cultivars	0.38 d	0.40 c	0.44 a	0.38 e	0.35 g	0.41 b	0.37 f	

The same letters, uppercase in the columns and lowercase in the lines, do not differ by the Tukey test (*P* ≤ 0.05).

In terms of the dose factor, the highest dose of 2 ml/L presented the highest average soluble solids content (6.51 °Brix), followed by the control (0 ml/L) and the 1 ml/L dose, with values of 6.20 and 5.88 °Brix, respectively. The sugar content of the studied cultivars increased with the application of the biostimulant at a rate of 2 ml/L (Table 7).

Regarding the titratable acidity, the statistical analysis indicated that there were significant differences in the varieties and doses of biostimulants and in the interactions between the two factors (Table 5).

On the other hand, for cultivars, the Festival had a higher average percentage (%) of citric acid (0.44 %), similar to that of Francesita (0.41 %) and Camino Real (0.40 %), but different from Sabrina and Sweet Charlie (0.38 %), early Bright (0.37 %) and the lowest value Dover (0.35 %).

Regarding the dose factor, the highest dose (2 ml/L) resulted in better performance, with an average of 0.39 %, surpassing the other doses.

Statistical analysis of pH revealed significant differences among cultivars, but not among biostimulant doses. A significant interaction between cultivar and biostimulant dose was also found (Table 5).

The pH values were between 3.2 and 3.4, with the highest average being 3.67 for the Sabrina Cultivar. Camino Real, Early Bright and Sweet Charlie obtained pH values of 3.58, 3.53 and 3.52, respectively, followed by Francesita con 3.46, Festival 3.4 and Dover 3.36, with the lowest values (Table 8).

**Table 8:** Hydrogen Potential (pH) and Firmness of fruits of strawberry cultivars based on biostimulant levels.

pH								
Biostimulant dose	Sabrina	Camino Real	Festival	Sweet Charlie	Dover	Francesita	Early Bright	$\bar{x}$ dose
0 ml L^-1^	3.7 A a	3.46 B c	3.45 A c	3.56 A b	3.37 A d	3.35 C d	3.56 A c	3.49 A
1 ml L^-1^	3.66 A a	3.65 A ab	3.42 A c	3.48 B c	3.31 B d	3.59 A b	3.44 A c	3.51 A
2 ml L^-1^	3.64 A a	3.63 A a	3.33 B d	3.53 AB b	3. 4 A c	3.44 B c	3.58 A ab	3.51 A
$\bar{x}$ cultivars	3.67 a	3.58 b	3.4 e	3.52 c	3.36 f	3.46 d	3.53 c	
Fruit firmness (Kgf cm ^-^^3^)								
0 ml L^-1^	0.67 B de	1.36 A a	0.59 A f	0.72 A d	0.65 B e	0.99 A b	0.87 B c	0.84 A
1 ml L^-1^	0.69 B c	0.47 C d	0.51 B d	0.75 A b	0.67 B c	0.83 C a	0.82 C a	0.68 C
2 ml L^-^1	0.74 A c	0.52 B e	0.58 A d	0.56 B de	0.85 A b	0.90 B e	1. 18 A a	0.76 B
$\bar{x}$ cultivars	0.7 de	0.78 c	0.58 f	0.68 e	0.72 d	0.90 b	0.96 a	

The same letters, uppercase in the columns and lowercase in the lines, do not differ by the Tukey test (*P* ≤ 0.05).

For the firmness variable, significant differences were found for cultivar, dose and the cultivar interaction with respect to the dose of biostimulant (Table 8).

In relation to the cultivars, Early Bright presented greater firmness (0.96 kgf cm^-3^), similar to Francesita (0.90 kgf cm^-3^), but different from Camino Real and Dover (0.78 and 0.72 kgf cm^-^^3^), Sabrina (0.70 kgf cm^-3^), Sweet Charlie (0.68 kgf cm^-3^), and the Festival, with the lowest average (0.58 kgf cm^-3^) (Table 8).

The dose control of 0 ml/L had the greatest effect on the firmness of the fruits, followed by the highest dose of 2 ml/L and finally that of 1 ml/L (Table 8).

Considering the interaction, for the Dover, Sabrina, Early Bright and Festival cultivars, a dose of 2 ml/L resulted in better values. However, for the other cultivars, the control (0 ml/L) resulted in greater firmness of the fruits (Table 8).

## 4 Experimental Design, Materials and Methods

### 4.1 Place of the experiment, climate and soil

The experimental work was carried out from May to August 2022, at Universidad Nacional of Asunción, Facultad de Ciencias Agrarias, located in the city of San Lorenzo, University Campus, at 25° 20′17′' south latitude and 57° 31′06′' west latitude and an altitude of 120 m above sea level.

**Fig. 2:**
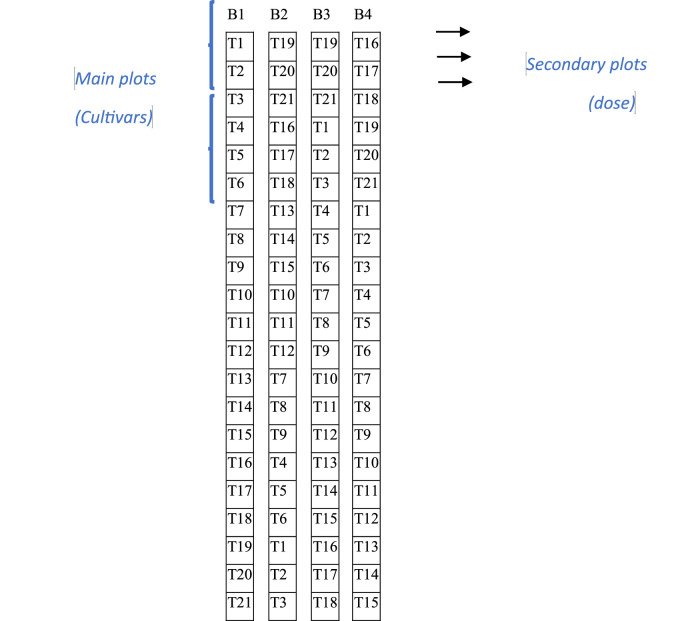
Distribution of the blocks and treatments used in the experiment.

The climatic of San Lorenzo is classified as warm and temperate, with an annual temperature range of 18 °C, to 29 °C and an annual average temperature of 24 °C. An average annual rainfall of 1400 mm (Fig. 2) characterizes San Lorenzo (Central department).

With respect to edaphic characteristics, the soil of the region is classified as a Rhodic Paleudult, has a considerable argillic horizon, reddish coloration, presents a udic regime of humidity, and is classified with the ultisol order (López et al. 1995) [18].

### 4.2 Treatments and experimental design

Seven varieties of strawberry were used: Dover, Sweet Charlie, Festival, Sabrina, Camino Real, Early Bright and Francesita and three doses of commercial biostimulant (topseed gram) (0 ml/L, 1 ml/L and 2 ml/L).

There were 21 treatments with four repetitions, which resulted in 84 experimental units. We worked with 32 plants per experimental unit distributed in four rows of eight plants within a plank, measuring 2.1 m wide and 2 m long (Fig. 2). The total population of plants was 2688, and they were cultivated in a total area of 231 m^2^. The pathways were 0.5 m wide.

In each block, each cultivar was designated as the main plot, and each dose was identified as a secondary plot, which in turn was found within each main plot (Fig. 2)*.*

The variables studied were total and commercial number of fruits, total and commercial mass of fruits, total and commercial yield of fruits and the post-harvest variables were: fruit length, fruit diameter, total soluble solids (°B), fruit shape, firmness, tiritable acidity and pH.

### 4.3 Experimental installation

#### 4.3.1 Soil analysis

Soil samples were obtained 30 days before the plots were prepared. The soil of the experimental area belongs to the ultisol order, and the chemical analysis carried out on the sample extracted from the experimental plot yielded the following results: pH = 5.90, organic matter = 0.87 %, P = 90.96 mg/kg, Ca +2 = 2.18 cmolc/kg, Mg + 2 = 1.38 cmolc/kg, K + = 0.07 cmolc/kg and Na = 00 cmolc/kg.

#### 4.3.2 Seedling transplant

Before transplantation, 150 µm thick bicolor (white/black) padding was placed on each plank.

The seedlings were transplanted with clods of earth previously prepared in plastic pots by a local producer. The planting distance was 25 cm between rows and 25 cm between plants.

#### 4.3.3 Bioestimulat

The trade name of the product used is topseed gram. According to the description of the treatments, the corresponding doses were applied from the beginning of flowering every 15 days until the end of the cycle. According to its original specifications, it contains L-amino acids and a supplement with synthetic hormones with AIA, GA3 and cytokinins. Formulated for seed treatment and foliar applications. Its composition consists of: water-soluble molybdenum (Mo): 8 %; water-soluble cobalt (Co): 1 %; water-soluble nickel (Ni): 0.1 %; water-soluble boron (B): 0.2 %; water-soluble IBA: 0.0015 %; water-soluble gibberellin A3: 0.0015 %; water-soluble cytokinin (N6-furfuryladenine): 0.0030 %; algae extracts; fulvic and humic acids; free L-amino acids.

#### 4.3.4 Fertirrigation

The irrigation system included a drip with a flow rate of 1 L/h corresponding to each dripper, with a distance of 20 cm between drippers, and two drip tapes were placed per plank with a separation of 50 cm between them.

For the fertirrigation process, a Venturi injection system was used to deliver a complete nutrient solution to the crop weekly through the drip irrigation system (Table 9). Applications began five days after transplantation, with the weekly dose divided into three separate applications throughout the week.

**Table 9:** Mineral elements and doses used for fertirrigation.

Fase	Nutrient				
	08-07-40 (g)	10-50-10 (g)	Magnesium sulfate (g)	Calcium Nitrate (g)	Vit-Org (ml)
Vegetative	8064	1350	1350	11080	5370
Reproductive	12.246	1755	3510	12246	4914
Total	20310	3105	4860	22326	10284

For postharvest variables such as fruit length, fruit diameter, total soluble solids, fruit shape, firmness, tiritable acidity, (methodology mentioned by Correa 2017) and pH, ten fruits with 100 % red color were randomly taken from each treatment.

## Limitations

None.

## Ethics Statement

The current work does not involve human subjects, animal experiments or any data collected from social media platforms*.*

## CRediT Author Statement

**Maria Celina Villagra Luraschi:** Conceptualization, Methodology, Research, Formal analysis. **Noelia Isabel Godoy Medina:** Conceptualization, Methodology, Research, Formal analysis, Software, Data review, Validation. Victoria Rossmary Santacruz Oviedo*: Research, Data review, Validation, Visualization, Original Draf preparation, Writing and editing. **Romina Burgos Rotela:** Research, Validation, Visualization. **Cipriano Ramón Enciso-Garay:** Supervision, reviewing.

## Data Availability

Mendeley DataDataset on the use of biostimulants in strawberry cultivation under tropical (Original data)
